# Involvement in teaching improves learning in medical students: a randomized cross-over study

**DOI:** 10.1186/1472-6920-9-55

**Published:** 2009-08-25

**Authors:** Adam D Peets, Sylvain Coderre, Bruce Wright, Deirdre Jenkins, Kelly Burak, Shannon Leskosky, Kevin McLaughlin

**Affiliations:** 1 Division of Critical Care Medicine, University of British Columbia, Vancouver, Canada; 2 Centre for Health Education Scholarship, Faculty of Medicine, University of British Columbia, Vancouver, Canada; 3 Office of Undergraduate Medical Education, Faculty of Medicine, University of Calgary, Calgary, Canada; 4 Department of Medicine, Faculty of Medicine, University of Calgary, Calgary, Canada; 5 Department of Family Medicine, Faculty of Medicine, University of Calgary, Calgary, Canada

## Abstract

**Background:**

Peer-assisted learning has many purported benefits including preparing students as educators, improving communication skills and reducing faculty teaching burden. But comparatively little is known about the effects of teaching on learning outcomes of peer educators in medical education.

**Methods:**

One hundred and thirty-five first year medical students were randomly allocated to 11 small groups for the Gastroenterology/Hematology Course at the University of Calgary. For each of 22 sessions, two students were randomly selected from each group to be peer educators. Students were surveyed to estimate time spent preparing as peer educator versus group member. Students completed an end-of-course 94 question multiple choice exam. A paired t-test was used to compare performance on clinical presentations for which students were peer educators to those for which they were not.

**Results:**

Preparation time increased from a mean (SD) of 36 (33) minutes baseline to 99 (60) minutes when peer educators (Cohen's *d *= 1.3; p < 0.001). The mean score (SD) for clinical presentations in which students were peer educators was 80.7% (11.8) compared to77.6% (6.9) for those which they were not (*d *= 0.33; *p *< 0.01).

**Conclusion:**

Our results suggest that involvement in teaching small group sessions improves medical students' knowledge acquisition and retention.

## Background

Peer Assisted Learning (PAL) is an efficient and effective way of preparing medical students for their future role as educators.[1] While initial studies reported that PAL was inferior to faculty assisted learning, [2,3] more recent studies suggest that in some situations learning outcomes may be comparable. [4-8] But what about the peer educators: do they benefit academically from involvement in teaching?

There is extensive data to suggest that when children serve as peer educators their academic performance typically improves.[9] By contrast, little attention has been paid to the effects of involvement in teaching on the learning of peer educators in medical school. Tang *et al*. [10] studied the effect of involvement in teaching on the attitudes towards sociocultural issues in medicine of 12 second year students. They found a significant change in three of ten outcomes studied. But preparation for teaching in this study included a three hour workshop on sociocultural medicine – so it was not possible to separate the effects of additional education from those of involvement in teaching. In a recent study, Wong *et al*. [11] found that 199 student selected to be peer educators had higher USMLE and GPA scores than a control group of non-peer educators. But this study is prone to allocation bias as students were selected to be peer educators.

In this study, our objective was to evaluate the effect of participation in teaching on the learning of medical students. We did not provide coaching or additional educational resources to peer educators, and randomly assigned peer educator duties to all students in the class. We also used a cross-over design to determine if any knowledge gains were content-specific or of a generalized nature.[12] We predicted that involvement in teaching would improve preparation and, consequently, learning outcomes.

## Methods

### Subjects and study setting

Our subjects were 135 first year medical students at the University of Calgary. We have a three year clinical presentation curriculum,.[13] within which the first two years consist of seven integrated systems courses. We use a combination of didactic and small group teaching during these courses and each clinical presentation is covered in at least one didactic and small group session. Our study was part of the formal academic curriculum for the Gastroenterology/Hematology Course, which lasted for 12 weeks and covered 16 clinical presentations spread over 22 small group sessions. The Conjoint Health Research Ethics Board for the University of Calgary and Calgary Health Region approved our study and written informed consent was obtained from participants.

### Small group learning

There are typically 14 students in each of our small groups and students remain in the same groups throughout the first two years of medical school. Our small group sessions are two hours long and focus on a single clinical presentation. Each session consists of one or more clinical cases with questions to stimulate discussion and highlight important teaching points. We give students the cases and questions in advance and expect them to prepare for the session. Attendance at each small group session is mandatory.

During our study each small group was led by two peer educators and a faculty preceptor. We instructed faculty to allow the peer educators to lead the discussion but to intervene to correct inaccuracies, reinforce and elaborate on important teaching points, summarize, and to ensure that the group covered all the material during the allotted time.

### Study design

This was a randomized cross-over study. Students were randomized to each of their small groups at the beginning of the academic year and for each small group session we randomly selected two students to be peer educators. We did not coach students prior to the sessions, or give them additional educational resources. We randomly allocated preceptors to small groups as we did not have the same preceptors available for each of the small group sessions.

### Assessment of student preparation and learning outcomes

In order to assess the impact that being a peer educator has on students' study habits, an anonymous email questionnaire was sent to all students asking them to provide an estimate of how much time they spent preparing for small group sessions when they were and were not peer educators. We subsequently divided the class into tertiles based on the amount of time they spent preparing for sessions when they were group members; low (0–15 minutes), moderate (16–59 minutes) or high (60 or more minutes). The purpose was to evaluate how assuming the role of peer educator may have differentially affected the study habits of those students who usually prepare for small groups (highest tertile) compared to those that tend not to prepare (lowest tertile).

We evaluated learning outcomes using a 94 item multiple choice question (MCQ) certifying examination administered at the end of the course. This evaluation had a blueprint linking each question to an objective and a clinical presentation.[14] While all clinical presentations taught were also examined, the number of questions per clinical presentation varied according to the amount of classroom time dedicated to the topic. The minimum performance level (MPL) for the examination was set using the modified Nedelsky method.[15] We used Cronbach's alpha to calculate examination reliability. We evaluated the performance of each student on each clinical presentation, and then calculated their mean score for clinical presentations in which they had, or had not, been peer educators.

### Data Analysis

Our analysis was based upon the principle of 'intention-to-treat'. We used a paired t-test to compare the amount of time students spent preparing as a group member to that spent as a peer educator. MCQ performance on clinical presentations for which students were peer educators to those for which they were not was also compared with a paired t-test. One-way analysis of variance (ANOVA) was used to compare the tertiles for mean time spent preparing for sessions when they were peer educators and also to compare the mean difference between the time spent preparing for sessions as a peer educator and as a group member. An effect size estimate for the difference in examination scores was calculated using Cohen's *d*, with appropriate technique for its use with paired data.[16] We performed our analysis using SPSS version 15.0.

## Results

One hundred and thirty-five students completed the course. Each student performed the role of peer educator 3 or 4 times during the Course. One hundred and nine students (72%) provided estimates of the time they spent preparing for small group sessions, while all 135 completed the MCQ examination.

**Figure 1:**
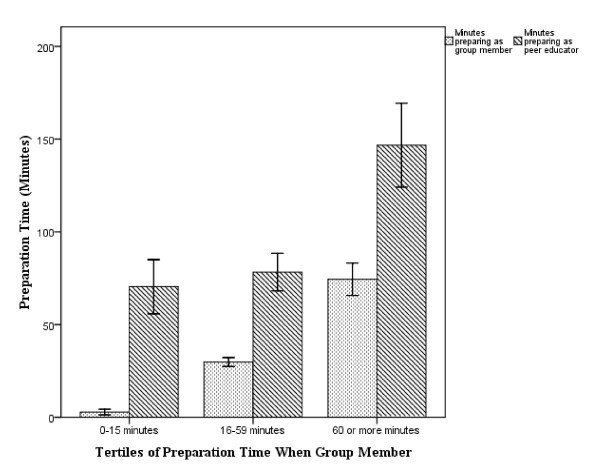
**Comparison of student preparation time based on tertiles of minutes spent preparing as group member (Error bars represent 95% confidence intervals)**.

Students spent a mean (SD) of 36 (33) minutes preparing for small groups when they were not peer educators, compared to 99 (60) minutes when they were (Cohen's *d *1.3, p < 0.001). A significant increase in preparation time was seen in each of the tertiles: from 3 (4) to 70 (42) minutes in the lowest (*d *2.2, p < 0.001); from 30 (7) to 78 (30) in the middle (*d *2.2, p < 0.001); and 74 (26) to 147 (68) in the highest (*d *1.4, p < 0.01). (Figure 1) Based on tertiles of preparation time as a group member, the high group spent nearly 75 minutes longer than the other two groups preparing for their role as peer educators (p < 0.001). When students in the lowest tertile assumed the peer educator role they increased their preparation time by a similar number of minutes as those in the highest tertile (67 vs 73, p = 0.7), and ended up preparing for the same number of total minutes as those in the middle tertile (70 vs 78, p = 0.37).

**Figure 2:**
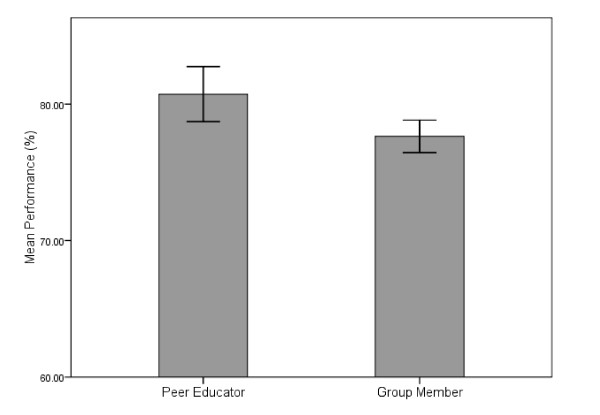
**Student performance on the multiple choice examination for clinical presentations based on educational group (Error bars represent 95% confidence intervals)**.

The reliability of the end of course multiple choice examination was 0.76. The overall MPL for the examination was 62.0%. The mean student score was 78.3% and 2 students were below the MPL. As students were randomly allocated to their teaching assignments, they were examined on between 16 and 22 MCQs for clinical presentations that they taught and 72 and 78 questions for clinical presentations where they were group members. Figure 2 shows the performance of students for clinical presentation in which they were, or were not, peer educators. The mean score (SD) for clinical presentations in which students were peer educators was 80.7% (11.8). This was significantly higher than clinicapresentations in which they were not peer educators: 77.6% (6.9), *p *< 0.01. The effect size for this difference was 'small to medium' (Cohen's *d *= 0.33).

## Discussion

We found when students assume the role of peer educator they prepare more for small group sessions, with the magnitude of change being greatest for those students who normally prepare least. We also found a small, but significant, improvement in knowledge acquisition and retention for clinical presentations where students had been peer educators compared to those for which they were simply a member of the group.

These findings lend support to the notion that medical student preparation for small group sessions improves learning. Several authors have advocated for PAL as a way to prepare medical students for their future role as educators. [17-19] PAL has been encouraged as a way to improve student communication skills, enhance motivation to learn, provide role models for junior students, and reduce faculty teaching burden.[17,19] The results of our study reinforce the importance of including improved learning on that long list of benefits.

Our results are consistent with those of other studies in the psychology and medical education literature that also found a learning benefit associated with involvement in teaching. [9-11] But compared to previous studies involving medical students, our study methodology was different. By virtue of its cross over design, so that each student served as their own control, as well as the random allocation of the intervention (involvement in teaching) and the lack of additional training we avoided the potential confounders of selection bias and performance bias of these previous studies.[10,11]

While it may seem intuitive that involvement in teaching should improve learning, this may not always be the case.[9] When a novice learner is presented with complex material a large intrinsic cognitive load is placed on working memory.[20] Performing tasks not directly related to learning – such as preparing for teaching – may generate an extrinsic cognitive load that may inhibit learning. Fortunately, we did not observe these potentially negative consequences of PAL – suggesting that this is a 'win-win' strategy.

In addition to increasing preparation time there are several explanations as to why involvement in teaching may improve the quality of learning. The first is that it motivates the learner to spend more time preparing, thereby possibly resulting in deeper learning. Teaching in the classroom also requires that concepts be verbally explained to the learner; this process of vocalization has been demonstrated to be an effective independent cognitive strategy for learning.[21] In addition, students may not be able to accurately assess their own knowledge deficiencies. By interacting with their peers in a classroom setting and attempting to answer questions from the group, peer educators may gain insight into which concepts they have a thorough understanding of and for which they need to study further. Finally, making participation in PAL an expectation may be enough to stimulate extrinsically motivated students to learn, while intrinsically motivated students may be rewarded by a sense of competence, autonomy, and relatedness by leading a small group discussion.[22] Although we did not evaluate this directly, irrespective of how much students typically prepared for small groups, we found that preparation time increased when they assumed the role of peer educator. Unfortunately, we were unable to assess whether the amount of time students spent preparing could be used to predict scores on the MCQ exam due to the anonymous nature of our survey, but should be explored further.

Importantly, our study design only allowed us to conclude students benefited from becoming involved in the entire process of teaching. Not only does this process include the time spent in the classroom actively teaching, but also includes the time spent preparing for the session and reflecting upon it after it has occurred. Determining to what extent each of these aspects contributed to the students' knowledge gains remains unknown. So while the act of teaching in the classroom may improve learning, the role that the significant amount of additional time students spent preparing for the session in our study cannot be ignored and should be explored in future studies.

There are several limitations to our study. Firstly, our results, while statistically significant, show a relatively small absolute difference in examination scores when a student was the peer educator compared to when they were participating as a group member. This may reflect that students still prepared for the sessions even when they were not peer educators or that the time between the small group sessions and the end of course examination was between one and 12 weeks – so our study evaluated the effects of involvement in teaching on knowledge retention rather than simply short-term knowledge acquisition. It should be noted that our effect size for knowledge gains is very similar to other studies in the literature that evaluated the effect of peer educators in courses of similar length.[9] Secondly, we conducted our study using a single course in a single medical school with a clinical presentation curriculum, so our results may not be generalizable. Finally, our use of student self-reported preparation times introduces the possibility of recall bias. Further studies are clearly needed to confirm, and explain, the performance benefits associated with involvement in teaching.

## Conclusion

PAL has traditionally been justified as a means of preparing students for their future roles as medical educators. But we now know that there are additional benefits to student involvement in teaching, such as improving communication skills and providing role models to junior students. Our results suggest that involvement in teaching also improves student performance, supporting an ancient Japanese proverb: "to teach is to learn".

## List of abbreviations

PAL: Peer Assisted Learning; USMLE: United States Medical Licencing Examination; GPA: Grade Point Average; MCQ: Multiple Choice Question; MPL: Minimum Performance Level; ANOVA: Analysis Of Variance; SD: Standard Deviation.

## Competing interests

The authors declare that they have no competing interests.

## Authors' contributions

ADP contributed to the conception and design of the study, acquisition of data, interpretation of data, drafting of manuscript and revising it critically for important intellectual content. SC contributed to the conception and design of the study, interpretation of data, drafting of manuscript and revising it critically for important intellectual content. BW, DJ, KB, SL and KM contributed to the interpretation of data, drafting of manuscript and revising it critically for important intellectual content. All authors read and approved the final manuscript.

## Note

At time of study ADP was a member of the Office of Undergraduate Medical Education at the University of Calgary.

## Pre-publication history

The pre-publication history for this paper can be accessed here:


